# Active RHOA favors retention of human hematopoietic stem/progenitor cells in their niche

**DOI:** 10.1186/1423-0127-20-66

**Published:** 2013-09-11

**Authors:** Bithiah Grace Jaganathan, Fernando Anjos-Afonso, Atul Kumar, Dominique Bonnet

**Affiliations:** 1 Haematopoietic Stem Cell Laboratory, Cancer Research UK, London Research Institute, 44 Lincoln’s Inn Fields, London WC2A 3PX, UK; 2 Department of Biotechnology, Indian Institute of Technology Guwahati, Guwahati, Assam, India

**Keywords:** RHOA, Hematopoietic stem cells, Stem cell migration, BM niche

## Abstract

**Background:**

Hematopoietic stem/progenitor cells (HSPCs) maintain the hematopoietic system by balancing their self-renewal and differentiation events. Hematopoietic stem cells also migrate to various sites and interact with their specific microenvironment to maintain the integrity of the system. Rho GTPases have been found to control the migration of hematopoietic cells and other cell types. Although the role of RAC1, RAC2 and CDC42 has been studied, the role of RHOA in human hematopoietic stem cells is unclear.

**Results:**

By utilizing constitutively active and dominant negative RHOA, we show that RHOA negatively regulates both *in vitro* and *in vivo* migration and dominant negative RHOA significantly increased the migration potential of human HSC/HPCs. Active RHOA expression favors the retention of hematopoietic stem/progenitor cells in the niche rather than migration and was found to lock the cells in the G0 cell cycle phase thereby affecting their long-term self-renewal potential.

**Conclusion:**

The current study demonstrates that down-regulation of RHOA might be used to facilitate the migration and homing of hematopoietic stem cells without affecting their long-term repopulating ability. This might be of interest especially for increasing the homing of *ex vivo* expanded HSPC.

## Background

Hematopoietic stem cells maintain the hematopoietic system through their proliferation, differentiation and migration from their niche. These processes are regulated at several levels and dysregulation was found to lead to pathological conditions. Of many factors that were found to control the migration, proliferation and the self-renewal capacity of stem cells, Rho GTPases also play an essential role. Rho GTPases belong to the Ras superfamily of GTPases. They are involved in the migration of different cell types, tumour cells and also in the differentiation of mesenchymal stromal cells [1-7]. Rho GTPases act as molecular switches that cycle between active GTP-bound state and inactive GDP-bound form. This conversion is tightly regulated by Guanine-nucleotide Exchange factor (GEFs) and GTPase-activating proteins (GAPs) [8,9]. Dysregulation of Rho GTPases was associated with neutrophil dysfunction, leukemia, and Fanconi anemia [10-12].

Enhanced activity of Rho GTPases was found to be associated with diverse hematologic abnormalities and malignancies [13-16]. Rac and Cdc42 have been found to be up-regulated in human AML [17,18]. Rac1, one of the members of the RhoGTPase family was shown to regulate the homing of hematopoietic stem cells through CXCR4/SDF1 signalling [19]. Rac1 was also found to be important for homing and engraftment of HSC into the BM niche and deficiency of Rac1 resulted in low engraftment rates [19]. Hematopoiesis was also found to be affected in Rac1 deficient embryo which is correlated with the impaired response to CXCL12 stimulation [20]. Hematopoietic specific Rac2 was found to be important for HSPC migration and adhesion and lack of Rac2 resulted in increased number of circulating HSPC. This suggests that Rac2 is essential for stem cell adhesion to its niche [21]. Furthermore, Rac2 was found to control these activities via the modulation of Rac1 and Cdc42 [22]. In addition, Rac2 mutation in hematopoietic cells resulted in severe neutrophilia [23,24] and decreased Cdc42 resulted in defective homing in Fanconi anemia [25]. Cdc42 was also found to regulate HSC migration and their retention in the hematopoietic stem cell niche. Mice lacking Cdc42 showed increased number of circulating HSC, rapidly cycling cells with deficiency in directional migration and adhesion [26]. Cdc42 activity also drastically affected the actin organization and cell adhesion. The increase in cycling of the cells was found to be mediated by p21^CIP1^ and c-Myc [26].

Although the role of Rac1, Rac2 and Cdc42 in hematopoiesis is well studied, function of RhoA is still not clearly understood. Our current study and other reports indicate that RhoA function seems to be cell type specific and species specific. In mice, inhibition of RhoA by dominant negative RhoAN19 reduced the migration of HSPCs but increased their engraftment [27]. However, the role of RhoA on human hematopoietic stem cell function, migration, and engraftment is still unclear.

Our studies in human cells show that RHOA inhibition increased the chemotactic migration of HSPCs without affecting their engraftment potential. We report here for the first time that RHOA expression favors retention of HSPCs in the niche rather than inducing their migration. Furthermore, inhibition of RHOA significantly increased the *in vivo* BM migration and homing of human HSPCs in mice without affecting their engraftment levels.

## Methods

### Isolation of lineage depleted cord blood mononuclear cells (HSPCs)

Umbilical cord blood was obtained after informed consent from the Royal London Hospital, London, UK in accordance with the local Research Ethics Committees guidelines. The mononuclear cells were separated by density gradient centrifugation and enriched for progenitor cells using human progenitor enrichment cocktail and Stem Sep column (Stem Cell Technologies, Vancouver, Canada). The resulting lineage depleted mononuclear cells (CBLin^-^) termed as Hematopoietic stem/Progenitor cells (HSPCs) were used for further experiments.

### Real-time PCR

Quantitative real-time PCR was performed on sub-fractions of the hematopoietic cells to quantify the transcript level of Rho GTPases RHOA. Total cellular RNA was extracted using RNeasy kit (Qiagen, Crawley, UK) and reverse transcribed into cDNA with Superscript III reverse transcriptase (Invitrogen). Real-time PCR was performed with SYBR-Green (ABI Biosystems, Carlsbad, USA) in an ABI 7900HT (ABI Biosystems) real-time PCR machine. The specificity of the product was verified in a 2% agarose gel. The primers used were: RHOA forward 5′-CTGGTGATTGTTGGTGATGG-3′ and RHOA reverse 5′-GCGATCATAATCTTCCTGCC-3′[28] and GAPDH forward 5′- GGGAAGGTGAAGGTCGGAGT-3′ and GAPDH reverse 5′- GGGTCATTGATGGCAACAATA-3′.

### Lentiviral vectors

The lentiviral vectors used for the study were based on pHRcPPT SIEW Sin vector with IRES regulating eGFP reporter gene. The vector contains SFFV (Spleen Focus Forming Virus)-LTR promoter and WPRE (Woodchuck Hepatitis Virus) element for post-transcriptional processing. RHOA constitutively active (RHOAV14) and dominant negative (RHOAN19) sequences were cloned from pBluescript vectors (kindly provided by Dr. Michael Way, Cancer Research UK, London, UK) by PCR using the primers F: 5′-GCGCGGATCCATGGCTGCCATCCGGAA-3′; R: 5′-GCGCGGATCCTCACAAGACAAGGCAAC-3′. The sequences were cloned into Topo Cloning vector (Invitrogen, Paisley, UK) and subcloned subsequently into SIEW by BamHI digestion. The orientation and the presence of mutation were confirmed by DNA sequencing. Lentiviral vector with only IRES GFP was used as experimental control.

### Lentiviral production and concentration

Lentiviral particles were generated by transfecting the transfer plasmid into 293 T cells with the packaging plasmids pCMVR8.94 and envelope pMD.G as described previously [3]. Viral supernatants were collected 48 and 72 hr after transfection and concentrated by ultracentrifugation.

### Lentiviral transduction of lineage depleted cord blood mononuclear cells

Freshly isolated or frozen lineage depleted mononuclear cells were stimulated for 8 hr with cytokines hFlt3L (50 ng/ml), hSCF (50 ng/ml), hIL-6 (10 ng/ml) and hTPO (20 ng/ml). After stimulation, transduction of HSPC cells were performed by the addition of the lentivirus particles containing control, RHOAV14 and RHOAN19 at a multiplicity of infection (M.O.I) of 80 in the presence of polybrene (4 μg/ml). Sixteen hours after transduction, the cells were washed and used for further experiments.

### Liquid culture, LTC-IC and CFU-C assay

CFU-C assay was performed for cells transduced with RHOA constructs in methylcellulose medium (Methocult H4434, Stem Cell Tech, Vancouver, Canada). Briefly, 1 × 10^3^ cells were seeded in 35 mm culture dishes and incubated at 37°C, 5% CO_2_. GFP positive cell aggregates of more than 50 cells were counted as colonies at 14 days in an inverted fluorescent microscope (Leica, Switzerland) according to the colony morphology. Long-term culture-initiating cell assay (LTC-IC) was performed by plating 1 × 10^4^ transduced cells on a monolayer of irradiated M2-10B4 cells and half-media replaced every 7 days. At the end of 5 weeks, the cells were collected, plated in methylcellulose medium for CFU-C assay and scored after 14 days. Liquid culture was performed to maintain the cells in progenitor stage by seeding the cells in serum free medium (Stem Cell Tech) containing hSCF (300 ng/ml), hFlt3L (300 ng/ml) and hTPO (20 ng/ml)and fresh media was added every 2–3 days.

### *In vitro* transwell migration assay

Cord blood lineage depleted cells (HSPCs) were transduced with control, constitutively active RHOA (RHOAV14) or dominant negative RHOA (RHOAN19) and cultured in serum free medium supplemented with hSCF (300 ng/ml), hFLT3L (300 ng/ml) and hTPO (20 ng/ml) for 7 days. Cytokines were added every 2–3 days. 100,000 cells were seeded in the transwell chambers (5 μm pore size) coated with fibronectin. SDF1α was added to the lower well (125 ng/ml) and the cells were allowed to migrate for 4 hours. The migrated cells in the lower well was collected and enumerated by flow cytometry (LSR II, Becton Dickinson) with the counting beads (Molecular Probes).

### Phenotype and cell cycle analysis

The transduced cells were identified by their expression of the reporter gene eGFP. Cell surface markers expression was determined by staining the cells with fluorescent conjugated antibodies and analyzed by flow cytometry. Cell cycle analysis was performed by fixing the cells with 2% paraformaldehyde and permeabilised with 0.1% Triton X-100. The cells were stained with anti-Ki67 conjugated with Alexa647 (Molecular Probes) and resuspended in 2% FCS containing DAPI (4′, 6-diamidino-2-phenylindole) and analyzed by flow cytometry.

### Short-term homing experiment

RHOA transduced HSPC cells were expanded in serum free media containing stem cell factor (SCF, 300 ng/ml), FMS likle tyrose kinase ligand (FLT3L, 300 ng/ml) and thrombopoietin (TPO, 20 ng/ml) for one week with addition of growth factors every second day. The cells were washed and 0.5 × 10^5^ transduced cells were injected intravenously in NOD/SCID/β2 mice. Bone marrow homing was analysed 24 hours post-injection by staining the bone marrow cells with antibodies against human CD45.

### *In vivo* migration and xeno-transplantation assay

All *in vivo* experiments were performed in accordance with the UK Home Office regulations and Cancer Research UK guidelines. NOD/SCID/β2 microglobulin null mice were purchased from Charles River Laboratories, UK. Mice aged 8–12 weeks were irradiated sub-lethally with an irradiation dose of 375 cGy from a ^137^Cs source. For migration experiments, 0.1 × 10^5^ transduced HSPCs cells were injected intra-bone into one of the hind limbs of the mice. Bones from each limb was collected separately after 7–8 weeks and analyzed for human cell engraftment by staining with antibodies against human CD45. For engraftment experiments, mice were intravenously injected with 0.5 – 1 × 10^5^ transduced HSPCs. The animals were sacrificed 12 weeks after transplantation, femurs and tibiae were collected and the cells were flushed and analyzed for human engraftment by flow cytometry.

### Statistical analysis

Statistical analysis was performed using One-way ANOVA test (SPSS software) and statistical significance in *in vivo* experiments were assessed using generalized linear models based on the negative binomial distribution (R software).

## Results

### Reduced RHOA levels in HSPC population

**Figure 1:**
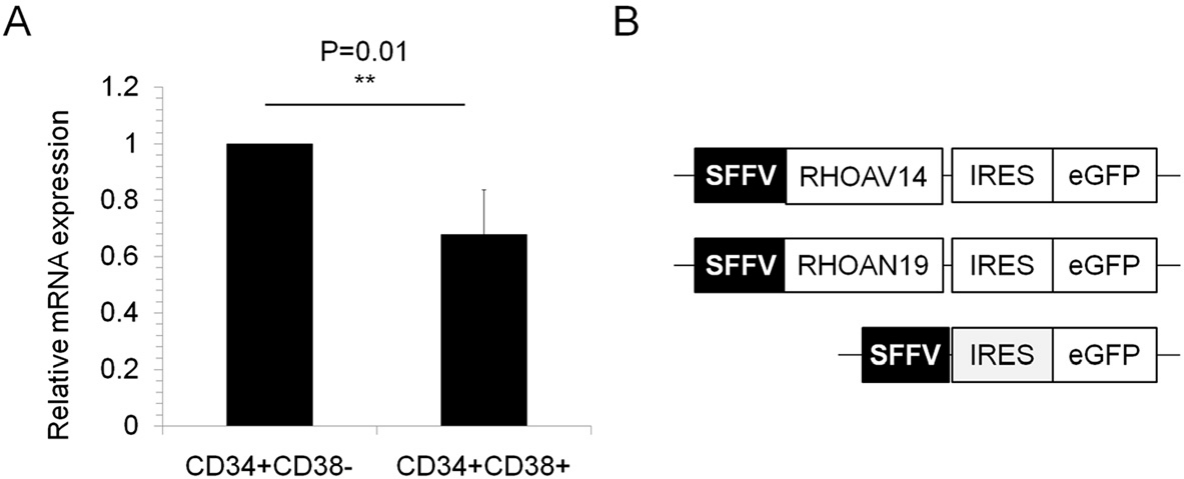
**RHOA endogenous and exogenous expression. (A)** Real-time PCR analysis to determine the mRNA levels of RHOA in lineage depleted cord blood hematopoietic sub populations CD34^+^CD38^-^ and CD34^+^CD38^+^ showed that RHOA was expressed in both the populations and it was significantly higher in CD34^+^CD38^-^ stem cell population. **(B)** Replication incompetent lentiviral vectors expressing RHOAV14 or RHOAN19 with SFFV (Spleen focus forming virus) promoter with EGFP (enhanced Green fluorescent protein) driven by IRES (Internal Ribosome Entry Site).

In order to understand the role of RHOA in maintaining the proliferation, migration and repopulating abilities of human hematopoietic stem/progenitor cells (HSPCs), we studied the expression of RHOA in CD34^+^CD38^-^ and CD34^+^CD38^+^ sub-fractions. Human cord blood derived mononuclear cells were sorted into hematopoietic stem and progenitor sub-populations based on markers such as CD34, CD38 and lineage markers. The mRNA level of RHOA in these fractions was determined by real-time quantitative PCR. RHOA expression could be found in CD34^+^CD38^-^ stem cell population and the level decreases as they start to differentiate into CD34^+^CD38^+^ progenitor cells (Figure 1A).

### Dominant negative RHOA increases HSPC migration

To analyze the effect of RHOA on hematopoietic cells, HSPCs were lentivirally transduced with constitutively active RHOA (RHOAV14), dominant negative RHOA (RHOAN19) and the control vector. The lentivirus used has an SFFV promoter expressing EGFP driven by IRES (Figure 1B). *RHOA* genes were cloned between the SFFV promoter and the IRES sequence. We found that RHOAV14 expression increased the RHOA active level whereas RHOAN19 expression effectively reduced the active RHOA level in the transduced cells.

**Figure 2:**
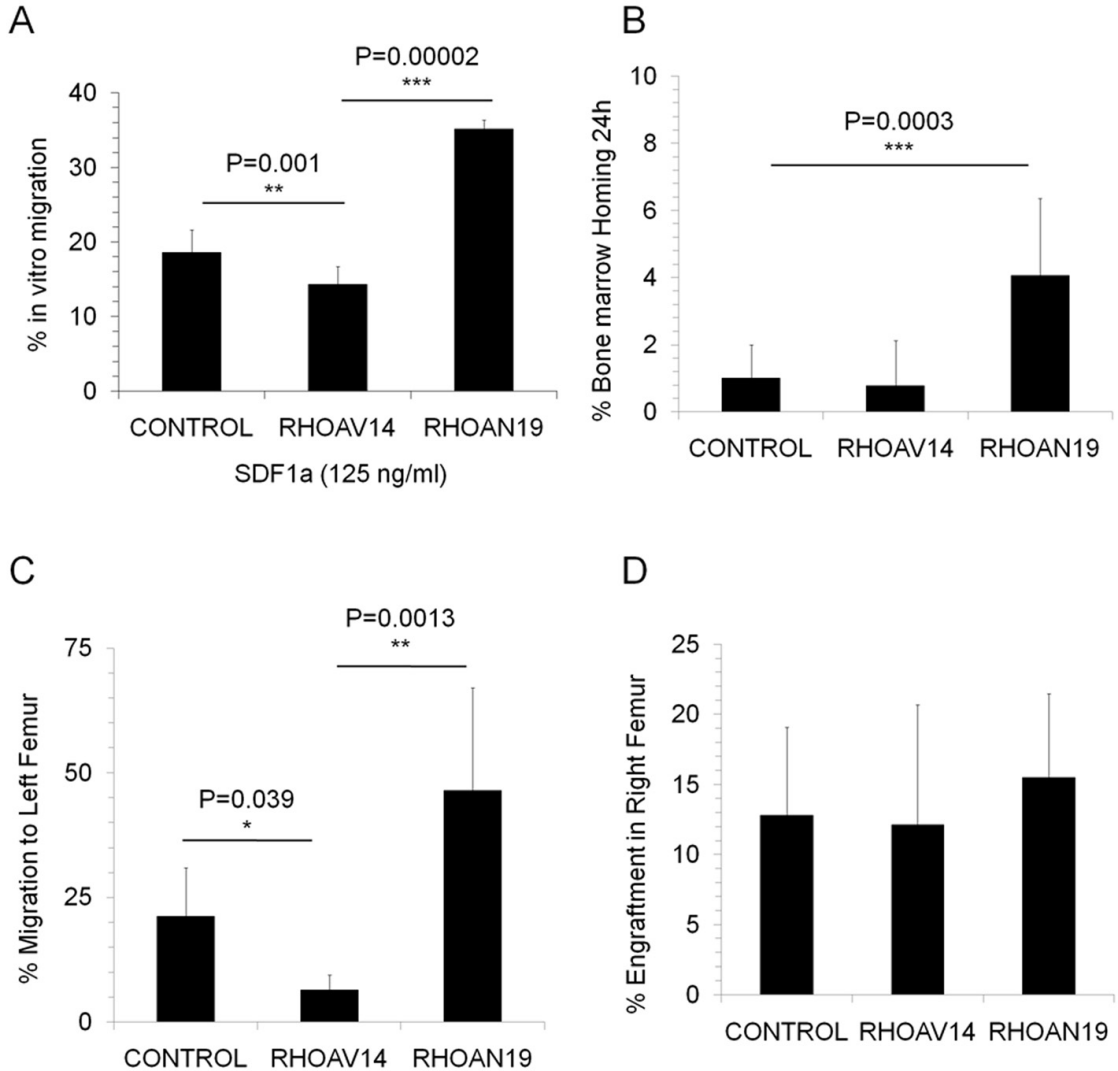
**In vitro and in vivo migration: (A) Migration of HSPC *in vitro*: Transduced HSPC were seeded onto the upper well of the transwell chamber and allowed to migrate towards SDF1α for 4 hours.** The percentage of cells migrated toward a gradient of SDF1α were calculated based on the absolute number of cells recovered in the lower chamber of transwell with respect to the total cells seeded. Values are mean ± SD, n = 4. **(B) *Short-term homing of RHOA modified HSPC*:** Transduced HSPCs were expanded in a liquid culture for 7 days containing cytokines. The cells were injected intravenously into NOD/SCIDβ2 microglobulin null mice and bone marrow was collected 24 hours post injection and the percentage homing was analyzed. Values are normalized to the control, Mean ± SEM, n = 3. **(C) *In vivo migration of RHOA modified HSPC*:** HSPC were transduced with active RHOA (RHOAV14) and dominant negative RHOA (RHOAN19) after stimulation with the cytokines for 8 hours. 24 hours after transduction, the cells were injected intra-bone into the right femur of the NOD/SCIDβ2 microglobulin null mice. Mice were sacrificed 7 weeks after injection, the left and right femur were collected separately. The percentage of engraftment in the right femur and left femur were analyzed separately by staining for human CD45 and GFP expression and the percentage of migration from right to the left femur were calculated. Values were normalized to the percentage of engraftment, mean ± SEM, n = 4-5. **(D)** Long-term engraftment of RHOA modified HPC was analyzed in a NOD/SCIDβ2 microglobulin null mice 7 weeks post intra-bone injection. Values are Mean ± SEM, n = 4-5.

To determine the effect of RHOA on the migration of HSPCs, RHOA transduced HSPCs were allowed to migrate in a transwell chamber towards SDF1α gradient. Post-migration analysis showed that there was a significant increase in the percentage of migration of RHOAN19 cells compared to the control group (35.5 ± 1.2% versus 18.6 ± 2.7%, p < 0.001) as shown in Figure 2A. Although increase in migration in HSPCs during RHOA down-regulation has been reported [2], there were no reports to suggest its role in *in vivo* migration. We hypothesized that decreased level of RHOA might also increase the homing and migration *in vivo*. To confirm this, we intravenously injected RHOA modified HSPCs into mice and determined the percentage of human (hCD45^+^ cells) present in the bone marrow 24 hours post-injection. We found that there was a significant increase in homing of HSPCs expressing RHOAN19 in the bone marrow compared to the control group (10.9 ± 2.7% in RHOAN19 versus 2.0 ± 1.9%, p = 0.0003, Figure 2B). No significant effect was observed when the constitutively active form RHOAV14 was over expressed in HSPCs (Figure 2A and B) probably due to a compensatory mechanism.

### Active RHOA favors retention in niche

**Figure 3:**
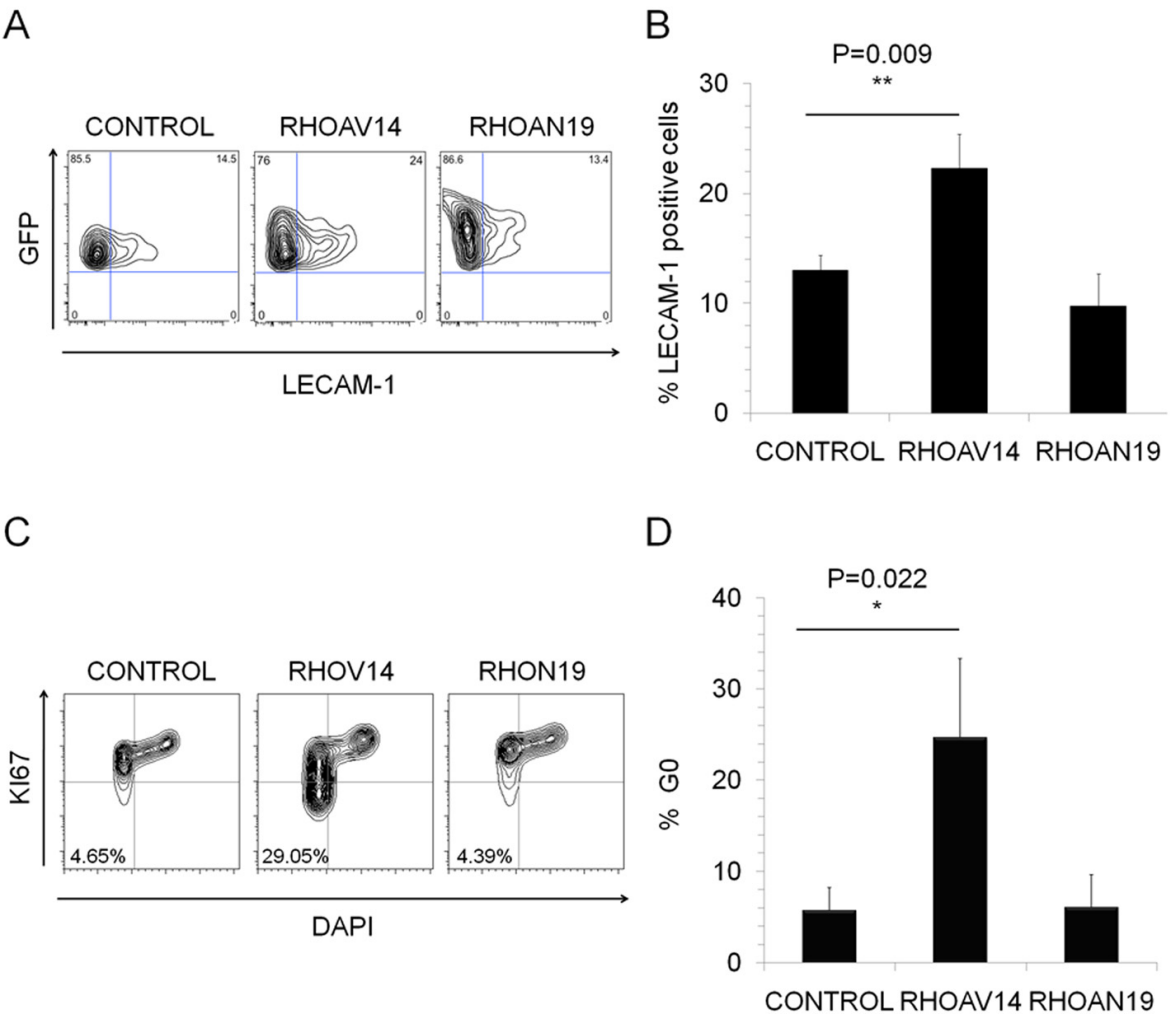
**Expression of CD62L and cell cycle. (A)** Flow cytometry analysis of CD62L expression in control, RHOAV14 and RHOAN19 HSPCs. Plots are a representative of at least 3 independent experiments. **(B)** RHOA transduced HSPCs were analyzed for their cell surface CD62L expression. Values are mean ± SD, n = 3 independent experiments. **(C)** RHOA modified HSPCs were cultured in liquid culture in the presence of cytokines and at day 7 were analyzed for their cell cycle profile using Ki-67 and DAPI. Plots are a representation of at least 3 independent experiments. **(D)** Graph representing the G0 percentage of control, RHOAV14 and RHOAN19 HPC/HSCs after 7 days in liquid culture. Values are Mean ± SD, n = 3 independent experiments.

To further confirm this and also to investigate the involvement of RHOA in adhesion and migration of HSPC in their niche, RHOA modified HSPCs were injected intra-bone in the right femur of the mice (injected bone) and the percentage of human CD45^+^ cells present in the non-injected femur was determined by flow cytometry 7 to 8 weeks post-transplant. We found that the percentage of engraftment was similar in all three groups (Figure 2D, control versus RHOAV14, p = 0.9585 and control versus RHOAN19, p = 0.284). However there was a 26% increase in the migration of RHOAN19 HSPCs from the injected (right) to the non-injected (left) femur of the mice compared to the control (Figure 2C), and there was a significant reduction in the migration of RHOAV14 expressing HSPC (Figure 2C, 21.10 ± 9.89% in control versus 6.46 ± 3.03% in RHOAV14, p = 0.039). To gain insight into the potential reason for changes in migration/homing potential of RHOA modified HSPC, we analyzed few adhesion molecules involved in the migration/homing. The integrin VLA-4 and VLA-5 expression was found to be similar in all cases (data not shown) however there was a significant increase in the expression of CD62L in RHOAV14 HSPCs compared to control cells (Figure 3A and B, 12.9 ± 1.4% in RHOAV14 cells versus 22.3 ± 3.1% in control cells, p = 0.009) but no decrease in the RHOAV19 HSPC group.

### Active RHOA decreases HSPC proliferation

**Figure 4:**
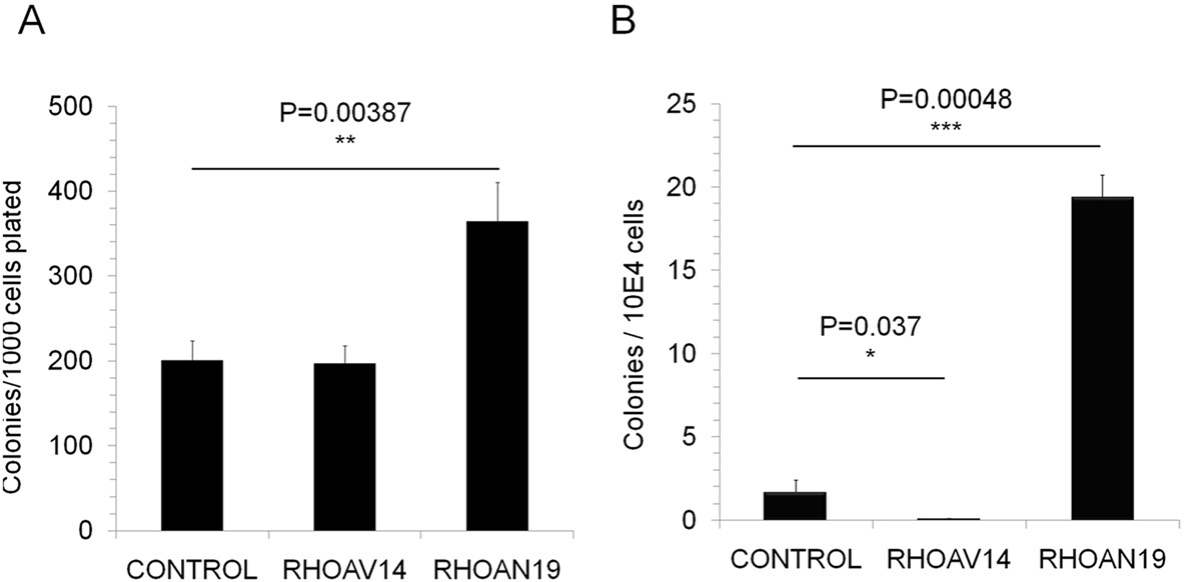
**Long and short term colony forming ability assay. (A)** Graph showing the number of colonies obtained per condition in a CFU-C assay when control, RHOAV14 and RHOAN19 cells were seeded in methylcellulose medium. Values are mean ± SD, n = 3; **(B)** Control, RHOAV14 and RHOAN19 HSPC were maintained in a long-term culture (LTC-IC)for 5 weeks and the colony forming ability was identified thereafter in a CFU-C assay. Number of colonies obtained per 10,000 cells are shown, values are mean ± SD, n = 3.

**Figure 5:**
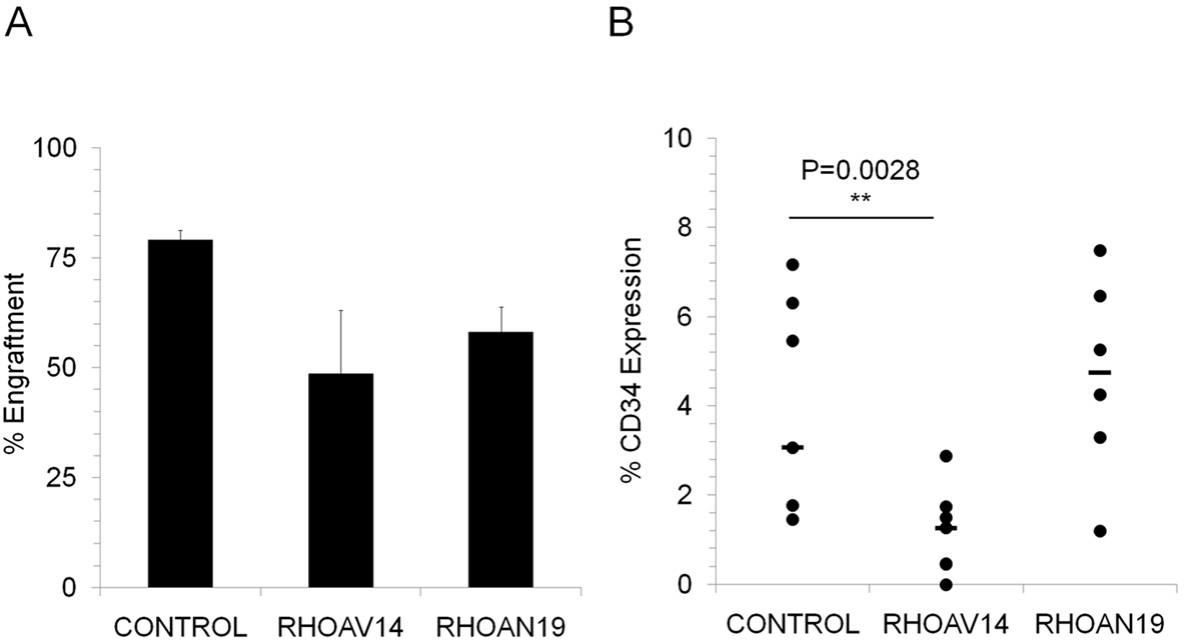
**Long term repopulation of RHOA modified HSPC. (A)** Percentage of human engraftment in Control, RHOAV14 and RHOAN19 expressing HSPCs, 12 weeks post-transplantation in immuno-deficient mice. Values are means ± SEM, n = 3. **(B)** Percentage of Human CD34 expression in Control, RHOAV14, RHOAN19 HSPCs 12 weeks post-transplantation. The percentage of CD34 expression was normalized to the percentage of engraftment. Each dot in the graph represents individual mouse and the bar represents the median value.

Since RHO GTPases have been found to control the cell cycle of non-hematopoietic cells, we studied the effect of RHOAV14 and RHOAN19 expression on the cell cycle status of HSPCs. Even after stimulation with cytokines, 5 fold more cells were found to be in G0 phase of the cell cycle in RHOAV14 expressing HSPC compared to control (Figure 3C, and D). No change in cell cycle was observed by expressing the constitutively dominant negative form (RHOAV19). To better understand if the difference in the cell cycle profile could affect the colony forming ability, we performed a CFU-C assay. No difference in the colony forming capacity of RHOAV14 HSPC was seen compared to control whereas a significant increase in colony number was observed in RHOAN19 group (Figure 4A). In order to evaluate the long-term effect of the modulation of RHOA, long-term culture initiating cells assay was performed. The number of colonies after LTC was reduced significantly in the RHOAV14 group whereas a significant increase was observed in the RHOAN19 group (Figure 4B). These results indicated that upon RHOAV14 expreession, the long-term but not the short-term colony forming ability of HSPCs was affected. To evaluate the *in vivo* effects of RHOA modulation, xeno-transplantation assay was performed. After 7–12 weeks post transplantation the percentage of engraftment in RHOA modified cells (RHOAV14 and RHOAN19) was comparable to the control group (Figure 5A). There was a significant reduction in the percentage of human CD34 positive cells in the RHOAV14 group compared to control (Figure 5B) implying that constitutively active RHOA significantly impede with the proliferation of HSPCs *in vivo*.

Taken together, these findings suggest that RHOA negatively regulates *in vitro* chemotactic and *in vivo* migration of human HSPCs and RHOA seems to favor the retention of HSPC in the niche rather than their migration. Active RHOA significantly reduced the proliferation of the HSPC *in vivo*.

## Discussion

Hematopoietic stem cell proliferation, self-renewal, differentiation and migration are controlled by various factors and stimuli. HSC migration to the bone marrow and its differentiation is essential to improve the HSC engraftment during transplantation therapy. Earlier studies have found that Rho GTPases, Rac 1, Rac 2, and Cdc42 control various aspects of migration, differentiation and self-renewal capabilities of murine HSPC but the role of RHOA in human hematopoiesis has not been investigated [19,21,22]. To understand the role of RHOA in human HSPCs, we modulated the active RHOA level in HSPCs through constitutively active and dominant negative forms. Transcript analysis of endogenous RHOA expression in the stem and progenitor cell population showed that RHOA was expressed in both stem enriched (CD34^+^CD38^-^) and progenitor enriched (CD34^+^CD38^+^) populations. In the present study, we show that decreased RHOA activity through dominant negative RHOA expression resulted in significantly increased *in vitro* migration through SDF1α stimuli and in *in vivo* migration and homing. We found a 2-fold increase in the migration of HSPC from the injected bone to the non-injected bone in xeno-transplantation assay. As previously reported in human HSPCs by Bug *et al.*[2] and in MSC by Jaganathan *et al.*[3], our present study suggests that decreased RHOA activity is essential for migration in human HSPCs both *in vitro* and *in vivo*. Our observations are in contrast with the results reported recently, where RhoAN19 expression resulted in the decrease of SDF1α induced migration in murine HSPCs [27]. In addition, the increase in RHOA level through RHOAV14 expression resulted in an increase in the retention of HSPCs in BM niche and a decreased migration to the contra-lateral non-injected bone in an *in vivo* migration assay although no difference in the engraftment percentage was seen. Our data goes along with an earlier report suggesting that migration and engraftment of HSPCs occurs as separate events *in vivo*[29] and thus might not always correlate together as suggested [30].

The cell cycle control by Rho GTPases has been documented well in hematopoietic progenitor cells, cancer cells and fibroblasts [27,31-34]. In murine hematopoietic cells, RhoAN19 expression resulted in decreased levels of P21Kip1/Waf1 and a higher number of cells in S phase [27]. Although we did not find any changes in the cell cycle profile when RHOAN19 was expressed in human HSPCs under cytokine induction, there was a significant increase in the number of colonies obtained in short-term and long-term colony forming assays, which shows that RHOA controls the proliferation of these cells. In addition, RHOAV14 expression in human HSPCs locked the cells in G0 phase of the cell cycle even under cytokine stimulation and reduced their long-term but not short-term colony forming ability or *in vivo* repopulating ability. Nevertheless, despite the non-significant effect on the 7 to 10 weeks long-term repopulation *in vivo*, we observed a significant decrease in the percentage of CD34^+^cells in the mice transplanted with RHOAV14, providing some evidence that RHOA negatively regulates the HSPC self-renewal but does not inhibit the progenitor cell proliferation. Studies using secondary transplantation should be performed to further confirm these data.

## Conclusions

In conclusion, we show that an active form of RHOA in human HSPCs reduces the *in vitro* and *in vivo* migration, bone marrow homing and lock the cells in G0 stage of the cell cycle, favoring the retention of the cells in the niche. In contrast RHOAN19 expression resulted in an increase in migration and in the short-term and long-term colony forming abilities without altering the cell cycle or the repopulating ability of these cells. Thus, down-regulation of RHOA might be used to facilitate the migration, homing of hematopoietic stem cells without affecting their long-term repopulating ability. This might be of interest especially for increasing the homing of *ex vivo* expanded HSPC.

## Competing interests

The authors declare that they have no competing interests.

## Authors’ contributions

BGJ, FAA, DB designed research, BGJ, FAA, AK performed research; BGJ, AK, analysed data, BGJ, FAA, AK, DB, wrote the manuscript. All authors read and approved the final manuscript.
